# Does quantification have a role to play in the future of bone SPECT?

**DOI:** 10.1186/s41824-019-0054-6

**Published:** 2019-05-03

**Authors:** James C. Ross, Dijana Vilić, Tom Sanderson, Stefan Vöö, John Dickson

**Affiliations:** 1 Institute of Nuclear Medicine T05, University College London Hospitals NHS Foundation Trust, 235 Euston Road, London, NW1 2BU UK; 2 Radiological Sciences Unit, Imperial College Healthcare NHS Trust, London, UK; 3 Institute of Nuclear Medicine, University College London, London, UK

**Keywords:** Bone scan, Nuclear medicine, Quantitative SPECT, Skeletal scintigraphy, SUV

## Abstract

Routinely, there is a visual basis to nuclear medicine reporting: a reporter subjectively places a patient’s condition into one of multiple discrete classes based on what they see. The addition of a quantitative result, such as a standardised uptake value (SUV), would provide a numerical insight into the nature of uptake, delivering greater objectivity, and perhaps improved patient management.

For bone scintigraphy in particular quantification could increase the accuracy of diagnosis by helping to differentiate normal from abnormal uptake. Access to quantitative data might also enhance our ability to characterise lesions, stratify and monitor patients’ conditions, and perform reliable dosimetry for radionuclide therapies. But is there enough evidence to suggest that we, as a community, should be making more effort to implement quantitative bone SPECT in routine clinical practice?

We carried out multiple queries through the PubMed search engine to facilitate a cross-sectional review of the current status of bone SPECT quantification. Highly cited papers were assessed in more focus to scrutinise their conclusions.

An increasing number of authors are reporting findings in terms of metrics such as SUV_max_. Although interest in the field in general remains high, the rate of clinical implementation of quantitative bone SPECT remains slow and there is a significant amount of validation required before we get carried away.

## Background

### Radionuclide bone scintigraphy

Nuclear medicine ‘bone scanning’ is employed to investigate active bone formation. Formation can result from a regular physiological process or be due to the presence of benign or malignant diseases. ^99m^Tc-labelled phosphate analogues are commonly used as radiotracers to visualise such processes, whereby the chosen bisphosphonate is adsorbed to the surfaces of hydroxyapatite crystals as it is cleared from soft tissue. Methylene diphosphonate (MDP), hydroxymethylene diphosphonate (HMDP) or hydroxyethylene diphosphonate (HDP), and 3,3-dicarboxypropane-1,1-diphosphonate (DPD) are the most common. Optimal visualisation is seen during ‘delayed phase imaging’ typically 2–4 hours subsequent to radiotracer administration. Bone scintigraphy as a diagnostic test is highly sensitive but not always specific (Van den Wyngaert et al. 2016).

There are numerous clinical indications for which skeletal nuclear medicine imaging is a valuable tool. The vast majority of conditions investigated can be classified as oncologic, inflammatory, rheumatologic, orthopaedic, infective, or metabolic. The cause of a patient’s symptoms may be known before imaging or it may be suspected or unknown. The combination and timings of planar (‘spot’ or whole-body), dynamic, and SPECT acquisitions should be selected according to the clinical question.

Uptake which is deemed suspicious in the planar view may necessitate a SPECT acquisition, which improves contrast and adds the advantage of being able to view radiotracer uptake in regions which are obfuscated by complex overlying and underlying anatomy. Increased specificity, positive predictive value, and additional diagnostic value following the introduction of SPECT-CT have been demonstrated in some applications, and it is currently recommended for patients with a high pre-test probability of metastases (Palmedo et al. 2014; Helyar et al. 2010; Hetzel et al. 2003; Schirrmeister et al. 2001). If SPECT is already planned as part of the acquisition protocol, more radioactivity should be administered to ensure the images exhibit sufficient image quality: in the UK, the Administration of Radioactive Substances Advisory Committee (ARSAC) diagnostic reference level is 800 MBq for when SPECT is included compared to 600 MBq for when it is not (Fraser 2018). Nuclear medicine physicians’ and radiologists’ interpretations of resultant image data are used to help the referrer answer the original clinical question(s), where the nuclear medicine report is corroborated against the findings of other investigations and drawn into an overall evidence-based conclusion.

Quantitative imaging may strengthen the corroborative process by instilling greater objectivity into the interpretations of nuclear medicine image data. So if there is sufficient evidence available to show that we can resourcefully provide quantification, we should feel compelled to roll it out into routine clinical practice.

### Quantitative SPECT

The purpose of a quantitative nuclear medicine measurement is to numerically assess, in relative or absolute terms, local concentrations of radiotracer and, if applicable, their wider distribution. Radiotracer uptake in bone represents two physiological activities: blood flow and the rate of bone formation. Pathological uptake, for example, can be recognised as caused by trauma from the increased local blood flow needed to supply the increased bone formation, where even small injuries to the bone can be seen on bone-scan images.

In principle, it is advantageous to numerically assess SPECT data over planar data because three-dimensional viewing reduces the unwanted count contribution in areas of interest from other sources and facilitates precise delineation of radiotracer uptake, whilst photon attenuation and scatter in the acquired data can be compensated for.

To make a judgement about patient’s conditions, a reporter must hypothesise subjectively what is causing high blood flow and high bone turnover, making a number of assumptions along the way. Quantification could help improve clinical reports by delivering numbers which directly relate to the uptake being visualised and reduce uncertainty. This may be of particular importance in differential diagnoses, when reporters are tasked with determining causes from several possibilities which entail different and even contradictory clinical management.

**Fig. 1:**
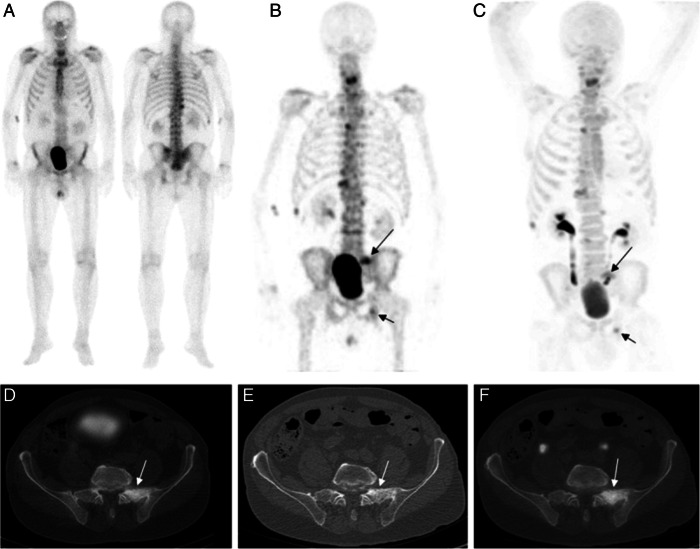
**a**–**f** According to one study, no statistically significant systematic differences were observed between results produced using planar whole-body bone scans, SPECT-CT, and PET-CT on newly diagnosed, high-risk prostate cancer (Fonager et al. 2017). But planar whole-body bone scans can lead to misclassifications. Fonager et al. described the following: ‘Anterior and posterior projections of the bone scan (**a**) were interpreted as non-metastatic on the dichotomous scale (with equivocal uptake in the pelvic region noted on the three-point scale). Both SPECT (**b**) and NaF PET (**c**) showed metastatic lesions on the maximum intensity projection images (long and short black arrows).’ (Images courtesy of e-Century PublishingCorporation, Wisconsin, USA)

The ultimate goal of quantitative SPECT, diagnostically, should be to help differentiate abnormalities (e.g. pathology, infection, spinal fusion, joint degeneration) from expected physiology and incidental uptake (Fig. 1), which could be facilitated by establishing numerical thresholds: if a numerical result fits within an expected range, it would give us more confidence in determining the nature of the uptake. Access to quantitative data might also enable us to better stratify a patient’s condition or monitor it, either in response to an intervention or to assess its stability, by providing a means of comparison. Meanwhile, sometimes, like in the case of classifying cancer, the reporting clinician just needs to know the presence of something, like metastases, but, in the case of bone SPECT, it can be the presence of abnormal activity which is difficult to determine.

### Metrics for osseous uptake

It goes without saying the results should be reliable. Numbers should be accurate, precise, and repeatable and they should hold clinical meaning and be condition-specific. Various metrics have already been explored for other applications and could be implemented for bone SPECT.

**Fig. 2:**
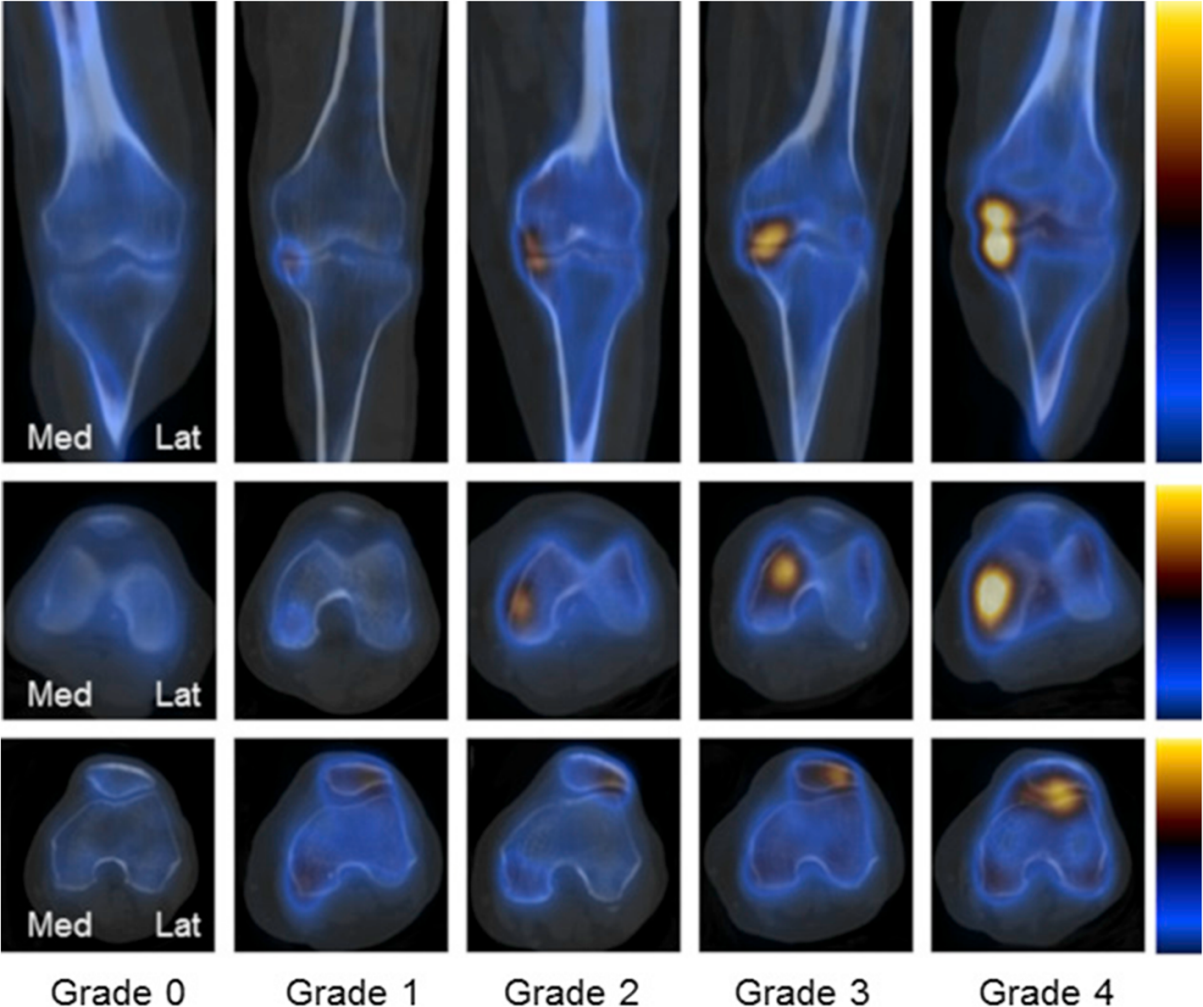
Kim et al. demonstrated a strong correlation between quantitative bone SPECT results and results from visual grading for patients with osteoarthritis of the knee (Kim et al. 2017). (Permission to reuse this figure was reached in agreement with Elsevier on 28th March 2019)

Visual grading to assign discrete numbers to uptake patterns is a semi-quantitative approach which can supplement clinical reports with numerical data (Fig. 2) (Al-Riyami et al. 2018; Kim et al. 2017). However, this is a subjective and crude method which does not always offer clinical value beyond what a written opinion already provides. Continuous numbers, on the other hand, provide something precise in nature.

The number of counts in a region by itself does not provide data which is specific to a patient’s body nor is it corrected for administered radioactivity. Its clinical utility is, therefore, greatly limited. Relative measures of uptake which incorporate counts in unaffected regions of bone as reference regions might factor out these patient-specific uncertainties. If an anatomical area of uptake can be proven consistent, like it has been shown for the liver in ^18^F-FDG (^18^F-labelled fludeoxyglucose) PET, a method could be standardised (e.g. to enable target-to-background ratios between pain-generating sites in abnormal and normal vertebrae in the lumbar spine to be generated) (Chirindel et al. 2015). The pelvis offers one such possibility (Wang et al. 2018).

Alternatively, results can be expressed in absolute terms of radioactivity concentration (Bq/cm^3^ or Bq/ml), which is useful for investigating avidity (e.g. for tumours). This approach, however, does not factor in the size of the patient. A standardised uptake value (SUV), normalised to a measure of body habitus, offers a preferable option. SUV was conceived for ^18^F-FDG PET but might play a role in locating pain generators in orthopaedic bone SPECT amongst other applications (Huang 2000). The average SUV across a whole volume, SUV_mean_, could be calculated, but the size and shape of the volume would depend on the choice of segmentation technique, be it manual outlining, thresholding, or something more sophisticated. SUV_max_, the maximum voxel value, could be used instead. It is the most-commonly used metric across SPECT and PET despite the fact that its reproducibility is dependent of the noise characteristics of the image, where a noisier image can result in a positive bias in the result. An alternative SUV metric which has been proposed in PET and which could be applied to quantitative SPECT is SUV_peak_ (Sher et al. 2016). Computing this entails expressing the maximum average voxel value within a 1-cm^3^ spherical volume, reducing the adverse influence of noise. However, this metric is often not available in commercial quantitative SPECT software.

If whole-body SPECT is available, metastatic bone burden (mean uptake × volume) might offer an additional insight into widespread disease by providing quantitative assessments of whole-body loads prior to and following radionuclide therapy (Umeda et al. 2018).

Ultimately, the choice of metric should be dictated by the clinical question.

## The current landscape

### Literature search

Using PubMed, we searched for published reports containing terms relevant to general quantitative nuclear medicine (Literature Search 1) on 3 January 2019. This returned 2144 results. A separate search (Literature Search 2) performed on the same date for quantitative bone scintigraphy specifically returned 76 results. Terms relating to the quantification of osseous uptake constituted the only inclusion criteria. Initially, 92 results were returned but 16 were subsequently omitted (1 duplicate, 1 non-nuclear medicine, 6 bone marrow, 1 kidney, 1 liver/spleen, 3 vascular, 3 cardiac).

**Fig. 3:**
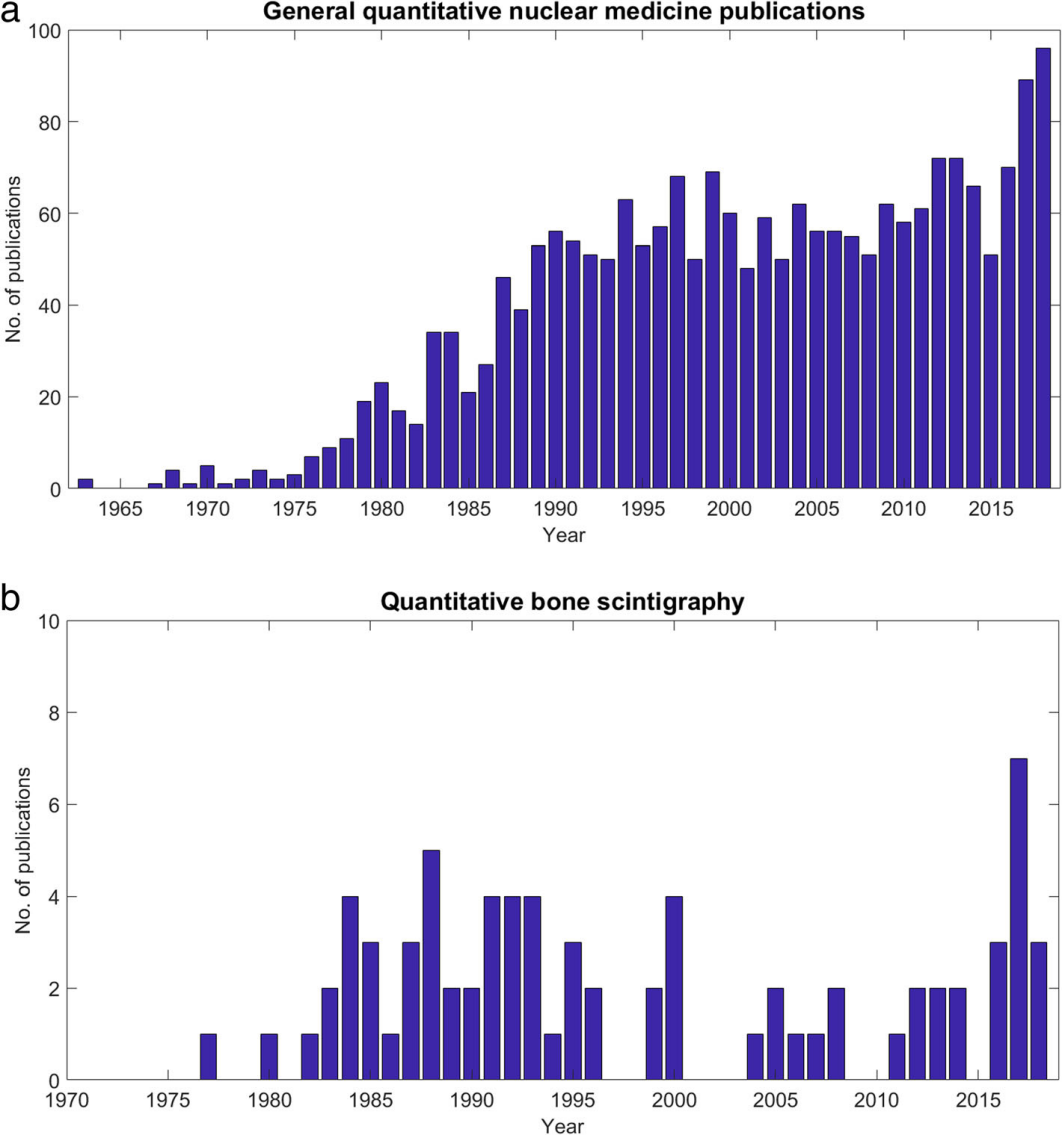
The results from Literature Search 1 illustrate a sustained rise in the number of general quantitative SPECT studies (**a**); the results from Literature Search 2 demonstrate that published data on quantitative bone scintigraphy is sparse (**b**). (Data reflect the numbers of papers published annually until the end of 2018)

Both sets of data are presented graphically in Fig. 3.

#### Literature search 1

(QUANTI*[Title] OR SUV*[Title] OR (STANDARDI*[Title] AND UPTAKE[Title] AND VALUE*[Title])) AND (SCINTIGRAPH*[Title/Abstract] OR SPECT-CT[Title/Abstract] OR SPECT/CT[Title/Abstract] OR SPECT[Title/Abstract])

#### Literature search 2

(QUANTI*[Title] OR SUV*[Title] OR (STANDARDI*[Title] AND UPTAKE[Title] AND VALUE[Title])) AND ((BONE[Title/Abstract]) OR (*SKELETAL[Title/Abstract])) AND (SCINTIGRAPH*[Title/Abstract] OR SPECT-CT[Title/Abstract] OR SPECT/CT[Title/Abstract]) AND (99 M[Text Word] OR DPD[Text Word] OR HDP[Text Word] OR HMDP[Text Word] OR MDP[Text Word] OR *PHOSPHONATE[Text Word])

A recent peak in the number of published studies under the umbrella of general nuclear medicine quantification is evident from Fig. 3. Higher usage emphasises the importance of caution. But, while there is sustained growth in general, there is scarcity in the number of investigations into quantitative bone scintigraphy.

### What authors are investigating

Table 1 documents results from Literature Search 2.

**Table 1:** Results from Literature Search 2, pertaining to 76 publications. Multiple criteria could be fulfilled by one study (number of publications in parentheses). ‘NM’ denotes ‘nuclear medicine’. Seven papers could not be found in full or translated

Indication	Study design	Metric	Analysis
Infective (0)	Animal research study (5)	Absolute (13)	Compared to non-NM data (32)
Inflammatory (6)	Cohort study (prospective) (35)	Kinetic (1)	Not compared to non-NM data (35)
Metabolic (3)	Cohort study (retrospective) (18)	Relative (39)	
Oncologic (16)	Descriptive (2)	SUV (9)	
Orthopaedic (13)	Systematic review (3)	Other (8)	
Rheumatologic (8)	Technical (6)		
Other (24)			

There have been various investigations into quantitative skeletal imaging over the last few decades but there is still a deficiency of useful evidence for any given application and for a given range of patient demographics. The most popular indication is oncologic (16/76). Yet even the evidence behind this application pertains to a range of primary tumour sites, ranging from prostate (8/16) to thyroid (1/16), while secondary sites, such as those in the lumbar spine, are explored non-specifically. Studies sprawl numerous other indications, with noticeable lacks of enquiry into bone infections (0/76) and metabolic diseases (3/10). This should be rectified if there are clinical needs to.

Only recently has there been a small pique in curiosity in the number of investigations into quantifying data from three-dimensional imaging. For instance, nine sets of authors have reported results with SUV, the first of which published data in 2013 (Cachovan et al. 2013). Yet, across all of the literature found, relative metrics, which involve no body habitus normalisation, were still the commonest (39/70).

In terms of study design, no prospective study has been reported since 2005, with 89% of this data being published before turn of the millennium. All investigations into SPECT SUV have been retrospective (9/9).

Just over half of the quantitative studies (35/67) contained no comparisons to non-nuclear medicine data. Independent data can be used to validate numbers through correlation analysis or direct comparisons to gold standards in the form of other image data, patient outcomes, or relevant biomarkers (e.g. bone mineral content, alkaline phosphatase level, prostate-specific antigen level). Corroborations with independent data are preferable but not always possible. Thirty-two studies documented such analyses.

### What authors say is abnormal

An issue is that numbers we can produce with relative ease do not inherently exhibit clinical significance. For a given examination, there are predominantly absences of what values constitute ‘normal’ and ‘abnormal’, preventing them from being clinically valuable. Benchmark values should be founded upon knowledge of uptake ranges for particular populations of patients. But, even when scans are performed on one gamma camera with the same acquisition protocol and reconstruction algorithm, there appears to be wide, inter-patient SUV ranges associated with the physiological or biochemical processes behind osseous uptake. This limits confidence in the numbers we might otherwise report with. It has been shown, for example, that SUV is a function of age, weight, and height with ^99m^Tc-MDP (Kaneta et al. 2016). A relationship between radioactivity concentration and bone mineral content ^99m^Tc-DPD has been demonstrated (Cachovan et al. 2013). Kuji et al. concluded the following (Kuji et al. 2017):

During aging, the inflammation and tissue remodelling in chondral tissue around bone leads to calcification and ossification. The different osteoblastic mechanism may affect SUV in prostate cancer with bone metastases and degenerative changes, reflecting the pathological osteoblastic nature of prostate cancer activity in higher SUV.

**Fig. 4:**
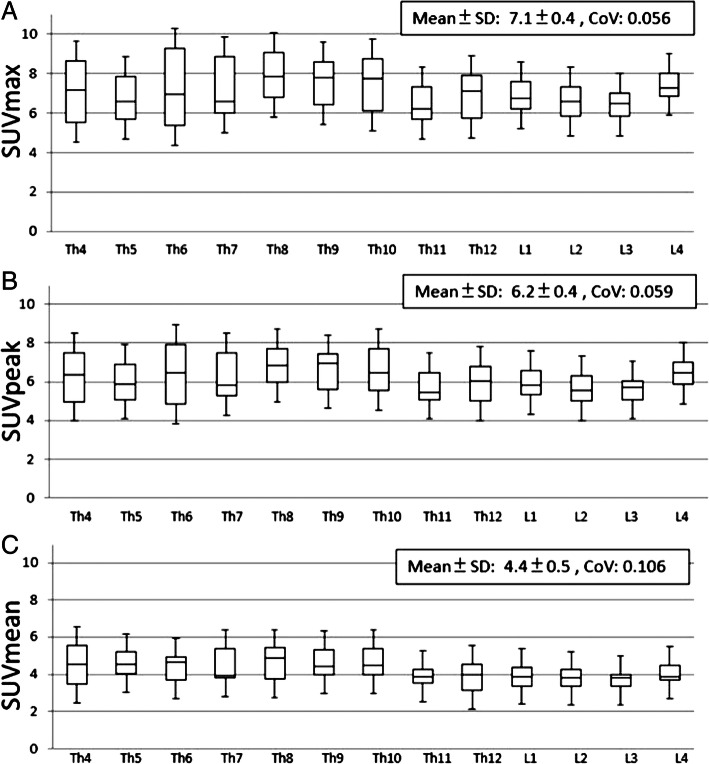
**a**–**c** SUV_max_ normalised to lean body mass has been reported as the best metric of osseous uptake for reducing patient variance (Kaneta et al. 2016). Labels on bottom-axes denote vertebrae. (Figure courtesy of e-Century PublishingCorporation, Wisconsin, USA)

SUV is, arguably, the most favourable metric of radioactivity concentration available to us—not least because its normalisation is to some expression of the patient’s body habitus, which facilitates comparisons between patients and between different imaging time points (e.g. for disease-monitoring). Although SUV was originally a construct designed to quantify avid areas of metabolism for ^18^F-FDG PET, there is no reason a similar normalisation of uptake to body habitus would not be beneficial for routine bone SPECT. However, there is no consensus on which expression of body habitus to use. Phosphate radiotracers are designed to accumulate in bone. Bone volume, therefore, is an obvious contender, where it could be calculated from the information seen on CT (e.g. the density and size of bones) and assumptions could be made from height and experimental data to facilitate this normalisation routinely. However, in one study, it was shown that SUV_max_ normalised to lean body mass is marginally better than SUV_max_ normalised to bone mineral content as well as other alternatives (Fig. 4) (Kaneta et al. 2016). So what do we use?

Regardless of the metric, expressing a bodily response to a particular trauma or pathology with a single number is reductionist in nature. The reality of generating a quantitative result which can be connected to a specific syndrome is complex, for a single number might non-specifically fall into one of a number of abnormal ranges for an array of conditions, and, given a lack of statistical precision, it could spuriously fall outside the correct one. For these reasons, the case is more compelling for follow-up quantification, which factors out some of these problems by enabling direct comparisons but still faces issues with quantitative precision and controlling what a patient does between scans.

The authors of one study investigated the utility of SUV in distinguishing bone metastases associated with primary prostate cancers from degenerative and normal areas for ^99m^Tc-MDP scintigraphy (Kuji et al. 2017). They put forward normal SUV_max_ ranges for vertebral bodies (7.58 ± 2.42 in the thoracic spine and 8.12 ± 2.24 in the lumbar spine), which were in line with previous studies (Kaneta et al. 2016; Cachovan et al. 2013). They were also able to differentiate metastases (40.90 ± 33.46) from degenerative changes (16.73 ± 6.74). Thus the authors claimed that they ‘prove that a discrimination of active bone metastasis can be established with high accuracy in patients with prostate cancer’ and that, despite a large spread on the results pertaining to metastases, ‘skeletal SUV can function as a reliable osteoblastic biomarker for discriminating active bone metastases with feasible accuracy’. However, the results are misleading: in order to characterise abnormal uptake, they only included SUV data for the three hottest lesions. A reporter still would have faced uncertainty when provided with any one lesion which was as intense in uptake. Additionally, the authors did not consider technical limitations nor did they attempt to characterise the unreliability of SUVs.

**Fig. 5:**
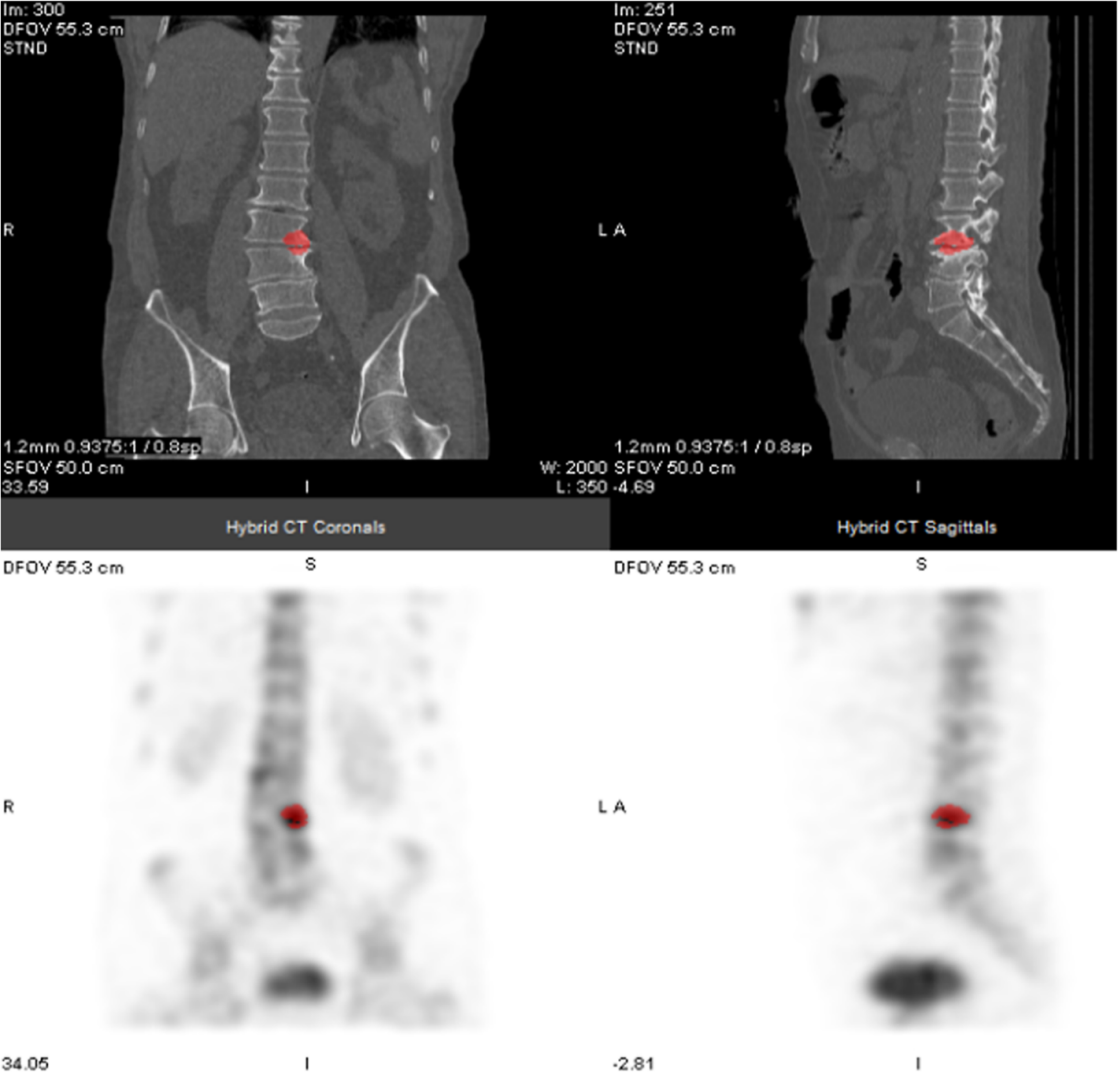
A local case study. Using GE’s Q.Volumetrix MI (GE Healthcare) the SUV_max_ at the confirmed site of fusion in the lumbar spine was found to be 28.7 (SUV_mean_ = 15.4), which sits comfortably above Kuji et al.’s normal range (but also within the range derived for metastases) (Kuji et al. 2017). The SUV_mean_ across all CT-segmented bone in the field of view was 3.48, which was lower than expected according to Kaneta et al.’s normal SUV_mean_ range (4.4 ± 0.5). Automated thresholding was employed to delineate the site. Is any of this more useful than visual interpretation? The quantitative result at the fusion site might have supplemented visual interpretation with something of prognostic value. In addition, comparisons could have been made upon follow-up SPECT-CT, enabling better ongoing characterisation of pain generation. Clearly, however, precision is still lacking

We applied normal ranges to a local case study and found physiological uptake to be somewhat quantitatively ambiguous (Fig. 5).

Uncertainty, regardless of the application, arises from factors related to the patient demographics, often resulting from an inherent variance in physiological and biochemical processes. This uncertainty represents the degree of confidence we should have in quantitative results. Imprecision, regardless of its cause, reduces the usefulness of numbers for supporting or excluding causes, classifications, or statuses that have been assigned to patients’ conditions visually.

## Obstacles

### Radiotracer dynamics

Nuclear medicine imaging is underpinned by the tracer principle: we attempt to understand a physiological or biochemical process (e.g. increased vascularisation to and bone remodelling around a tumour) which is not readily observable by studying the behaviour of a radiotracer acting as an agent within it. Ordinarily, this is interpreted by visually assessing the intensity or extent of focal or systemic radiotracer uptake in the images. But for a reporter’s opinion to bear relation to the clinical truth, an assumption has to be made that the level of uptake is proportionate to the physiological or biochemical process being traced. But is it?

If we cannot provide empirical evidence for the reliability of numbers used for clinical reporting, we can only operate with the hope that the numbers are anchored to the nature of the patient’s condition, when sometimes it is, based on some evidence, and when sometimes it is not, when quantitative imaging is fundamentally flawed. A significant amount of work is required by the scientific community to generate evidence for the former. Unlike the case for PET, demonstrable clinical benefit has not been observed in longitudinal or cross-sectional form for quantitative SPECT yet (Al-Riyami et al. 2018; De Laroche et al. 2018). Assessments of correlations between quantitative results and certain clinical outcomes would provide results that throw the most weight behind quantitative SPECT’s cause (e.g. degree of pain generation and time to next medical intervention, SUV_max_ of suspected infection site and correct diagnosis, metastatic bone burden, and survival or quality of life).

According to guidance from the European Association of Nuclear Medicine (EANM), factors to take into account when reporting bone SPECT images include localisation, intensity, size and shape, and number of lesions found (Van den Wyngaert et al. 2016). Quantification of uptake can supply this information through segmentation or thresholding, while results can be corroborated against other quantitative findings. However, owing to feint and small areas of uptake, segmentation is not always easy. Meanwhile, a distribution of the SUV values across a lesion could bear diagnostic information. For example, some bone lesions have a distinct increase in SUV on the edge of the lesions and a decreased SUV in the middle (e.g. in case of osteonecrosis) and vice versa (e.g. metastases). Quantification could, therefore, be incorporated into the current reporting style and complement current guidance if found to be useful. First, though, we need to understand how variance between patients is linked to the physiological or biochemical processes of radiotracer uptake we are studying so we are able to express it in our results, similar to how the influence of tissue fraction on lung uptake has been studied and accounted for in PET (Holman et al. 2015). Kinetic modelling of the radiotracer process would help us understand what results really mean by quantifying uptake time and clearance rates. Through kinetic modelling, we could learn how to mathematically account for the different ‘compartments’ in our models of radiotracer dynamics to understand the relationship between blood flow, bone remodelling activity, and patients’ conditions.

Quantitative SPECT is not fundamentally flawed; rather, the take-home message is this: without an understood context, a number is just a number and the greater the uncertainty, the greater the mask on quantitative bone SPECT’s potential.

### Technical limitations

If a cost-effective clinical benefit has been demonstrated for a technique elsewhere, it is almost a scientific duty to evaluate and implement it locally. The issue here is that such a benefit has not yet been demonstrated for the use of quantitative bone SPECT data.

This is not to say we are unable to evaluate it as a community. Technologically, the clinical nuclear medicine community is, arguably, prepared to at least investigate it. With a gamma camera capable of performing SPECT-CT and particular commercial software available to a department it could become routine without much technical difficulty—and many departments are now equipped with such capabilities.

**Fig. 6:**
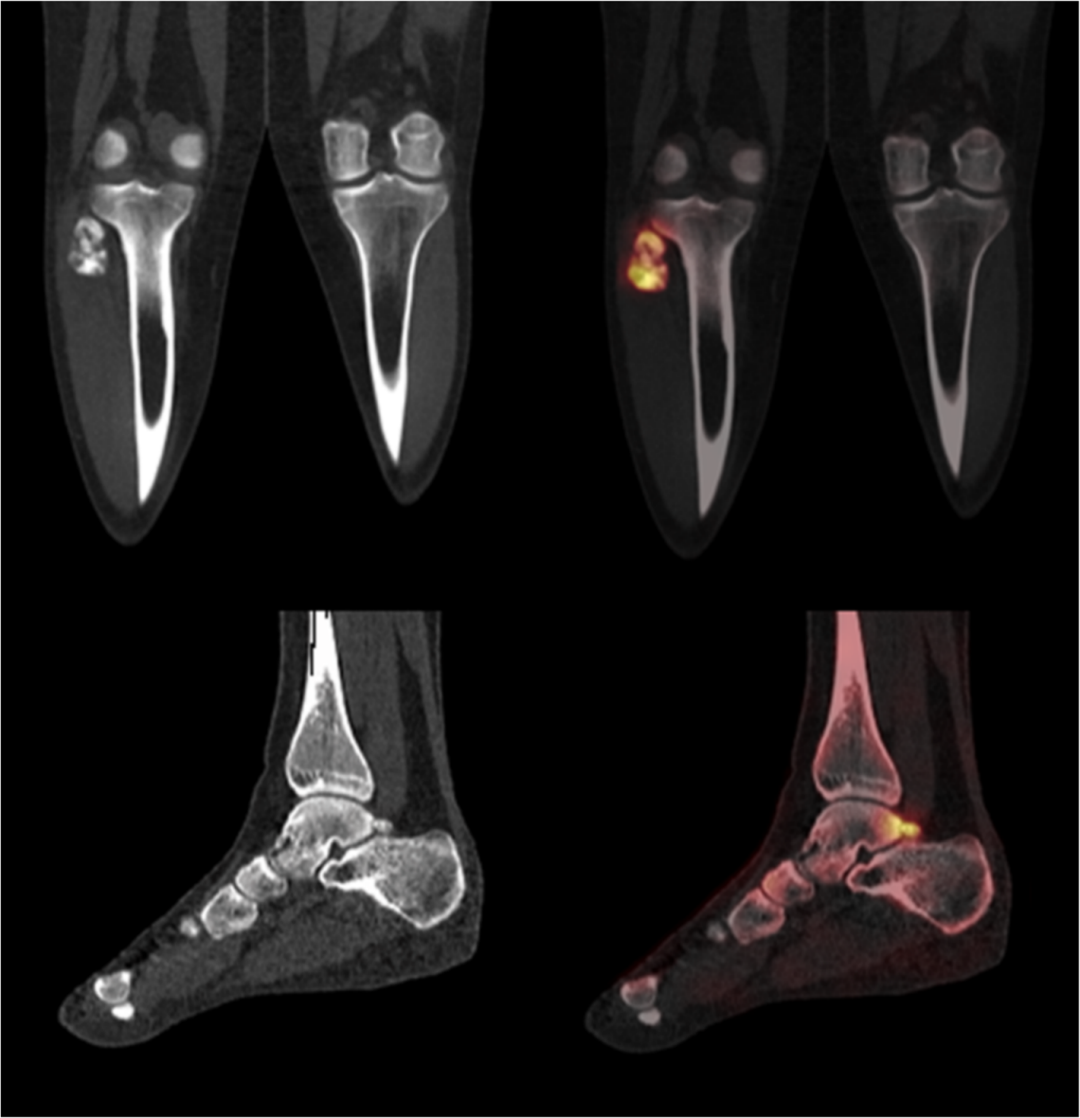
Images of an 18-year-old male’s right leg produced with xSPECT Quant™ and xSPECT Bone™ (Siemens Healthineers), containing a giant cell tumour in the proximal fibula. Uptake in proximal tibio-fibular joint was concluded as normal as it was mild and reactive and no erosion could be seen. Siemens claim that xSPECT Bone™ offers improved image quality and lesion localisation through sharper edge delineation. But is there enough evidence to assume it can be routinely called upon to perform accurate and reproducible quantification to support important clinical decisions? (Images courtesy of Siemens Healthineers, Erlangen, Germany)

**Fig. 7:**
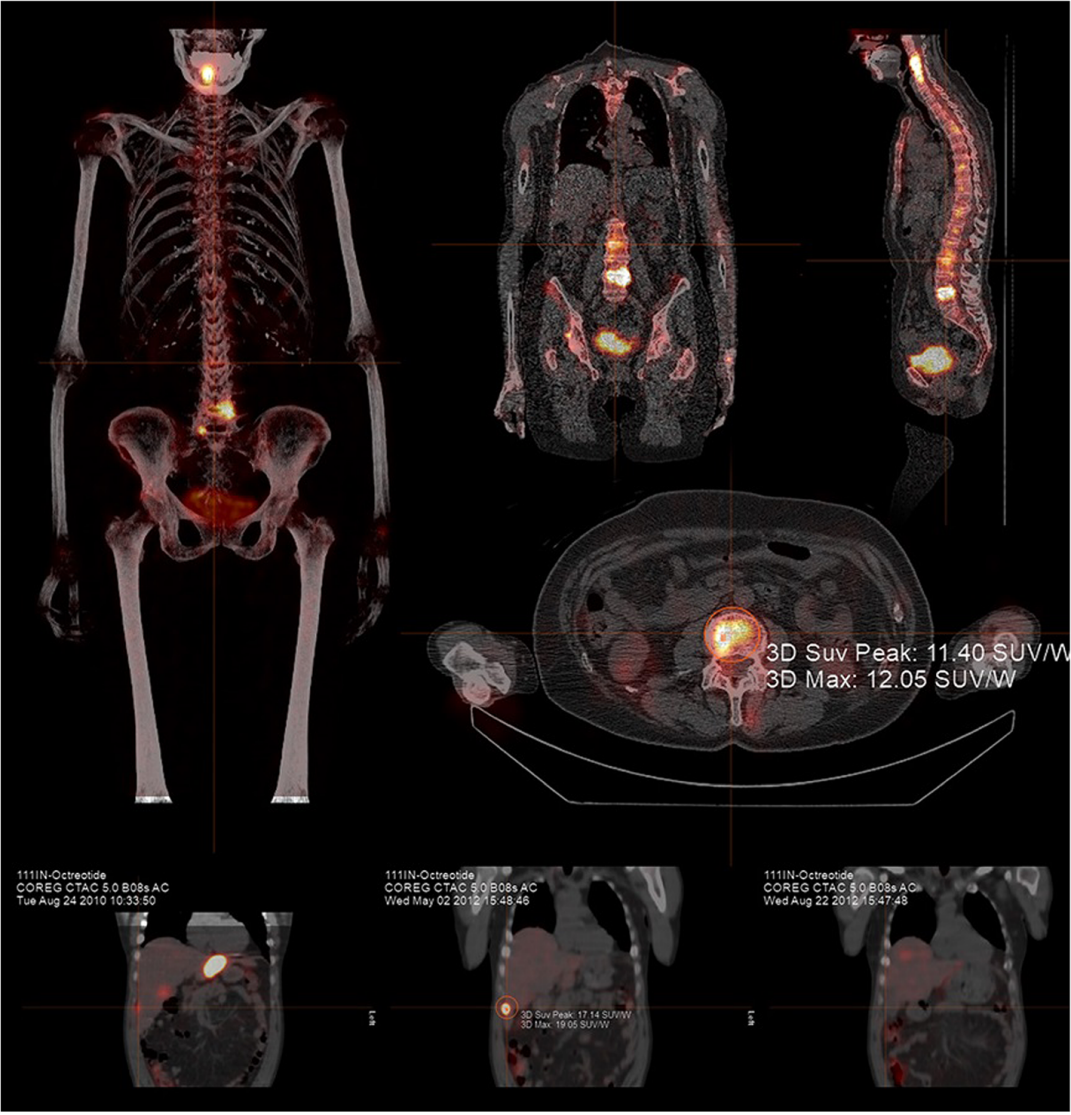
Absolute quantification is possible with Hybrid Recon™ (Hermes Medical Solutions). An SUV_max_ of 12.05 and an SUV_peak_ of 11.40 were generated in the delineated area of this patient’s lumbar spine. (Images courtesy of Hermes Medical Solutions, London, UK)

Vendors such as GE Healthcare (Fig. 5), Hermes Medical Solutions (Fig. 6), MIM, Osirix, and Siemens Healthineers (Fig. 7) now commercially offer various software packages which are furnished with quantitative tools. Their dedicated applications provide users with apparatus at the graphical-user-interface level which allow the user to express uptake in drawn volumes of interest. Each chosen platform boasts its own pros (e.g. Monte Carlo models for compensations) but impart its own cons (e.g. cost). Each department must weigh the financial cost against not only their present clinical need but also envisioned benefit.

Numerical results of quantitative imaging are intrinsically linked to image quality (Buchbender et al. 2013). In PET, it is known that quantitative results can suffer from unexpected but understood effects (‘artefacts’), including but certainly not limited to the influences of different uptake times, blood glucose levels, uptake in brown fat, and partial-volume effects. Nevertheless, authors have been able to show that numbers can still exhibit clinical value (Choi et al. 2018). In bone SPECT specifically, quantitative uncertainties can arise because of radiotracer-drug interactions (e.g. with iron supplements), metal-induced artefacts from prostheses influencing computed uptake values, dependency on hormone levels (e.g. oestrogen), and unknown rates of uptake and clearance from the blood, amongst other factors. The system resolution, acquisition time, and reconstruction conditions, such as the number of iterations and subsets applied for an iterative reconstruction algorithm like ordered-subset expectation maximisation (OSEM), image filtering, scatter correction, and the method and parameters of attenuation correction, can also influence results (Chicco et al. 2015; Tsujimoto et al. 2018).

Partial-volume effects for ^99m^Tc SPECT, if left unaccounted for, can cause objects with diameters of less than around 2 cm to exhibit reduced contrast, which applies to many bone lesions. A volume-specific recovery-coefficient correction can be derived from a recovery curve pertaining to a phantom containing a range of different volume inserts of known activities to mitigate this (Sanderson et al. 2015). However, this type of correction is currently not often implemented in commercially available software. Furthermore, it is not simple to correct for object size, shape, and uptake intensity, for these are properties which we are trying to determine.

Resolution modelling, which is included as standard in some commercial quantitative SPECT applications, is another option but can result in edge-enhancement artefacts, particularly at high iteration numbers, which will impact on segmentation quantitative accuracy (O’Mahoney and Murray 2013). The ‘Gibbs ringing’ that results is a response to sharp changes in uptake and disproportionately affects small regions of uptake, introduces wide uncertainties, and interferes with the interpretation of radioactivity concentration, particularly SUV_max_. Furthermore, the spatial resolution of SPECT is relatively large and might be a fundamental stumbling block which prevents quantification being of considerable benefit for focal uptake, whilst there is also quantitative bias, systematic differences between quantitative estimations and true values, to consider (Bailey and Willowson 2013; Armstrong and Hoffmann 2016). In terms of noise and image contrast, current guidelines do not contain recommendations for implementing dose administration protocols for bone scintigraphy to achieve similar image quality between patients nor is there evidence to suggest that we should.

All of these contributing factors to varying image quality within and across patient cohorts threaten standardisation and harmonisation. The IPEM Nuclear Medicine Software Working Party conducted a UK-wide audit of the quantitative characteristics of various SPECT reconstruction software packages (Jarritt et al. 2002). The findings, published in 2002, demonstrated striking differences in numerical results generated when supplied with the same projection data—even, in some cases, when comparing results produced with different revisions of the same software. It remains to be seen whether manufactures have resolved these inconsistencies. Combined with other factors which contribute to the uncertainties of radiotracer uptake accuracy and location, caution is recommended. Careful validation should be undertaken prior to the clinical application of a chosen technique. Its importance is emphasised by known inconsistencies between visual and quantitative interpretations (Beck et al. 2016; López Buitrago et al. 2017).

Nonetheless, a lot has changed in 17 years: progress is already underway and potential solutions are available (Nakahara et al. 2017; Miyaji et al. 2017; Vija 2013). Meanwhile, accuracy, in theory, is improving with time. Scatter and attenuation correction has come on markedly. Solid-state gamma cameras promise to increase sensitivity and improve energy resolution. Areas of tissue can already be semi-automatically segmented, not merely manually drawn, with more-developed techniques, where computational procedures such as seeding, interpolation, and thresholding are routinely available. However, question marks remain over the precision of our results, particularly for when dealing with oftentimes-ambiguous uptake in bone.

Such technical limitations might be mitigated in the future. The use of increasingly sophisticated methods of compensation for the partial-volume effect, for instance, may improve the accuracy of corrections as they are made specific to the spatially variant resolution of a particular system and its collimator-detector responses. Meanwhile, Monte Carlo models can be applied to estimate photon scatter contributions more accurately (Fig. 7). Currently, prevalent methods of compensation are underpinned by blunt energy-discrimination deduction.

We still face technical limitations which hamper our accuracy and precision, and, still, we have no true grasp of what numbers mean across various diseases and uptake intensities. Commercial applications attempt to provide solutions—but will this lead to the benefit of patient management?

## So is there a future?

From a technological perspective, clinical nuclear medicine departments, especially larger ones, often do have access to the required software to perform quantitative measurements. With the positive trend in quantitative SPECT continuing, the question for those interested in quantitative bone SPECT becomes where should we direct our focus? In 2016, EANM summarised the current status of quantitative bone SPECT as follows (Van den Wyngaert et al. 2016):

*Quantitative bone SPECT/CT is a novel technique with potentially useful applications in treatment response monitoring in bone. However, the exact role in routine clinical practice has yet to be determined.*

In fairness to opponents to change—usually pessimists and late adopters—they are debatably justified in pointing to current hurdles when resisting the implementation of quantitative SPECT: required resources are not always immediately available and workers might be willing but lack skill, knowledge, and managerial power. Is investigating this specific application ethical, given the current financial climate and other pressures on healthcare services? At what cost do we attempt to find out? Should we be focusing our resources elsewhere? While it is possible to explore the cost-effectiveness of more-routine applications of quantitative SPECT, we still need to test the water for skeletal scintigraphy (Stokke et al. 2017). Without compelling and substantial evidence of clinical benefit for a department’s particular fields of interest, and given demands on resources, not least staff time, it is difficult to grant quantitative bone SPECT enough impetus to render it a local and communal priority. With it, we could dutifully expedite its implementation.

Take the following case. We might be inclined to initiate drive towards metastatic bone burden reporting—say, in the case of ^223^Ra-Cl_2_ and ^177^Lu-PSMA (^177^Lu-labelled prostate-specific membrane antigen) for prostate cancer—such that we can monitor the whole disease prior to and following radionuclide therapy. Benefit has already been shown for whole-body images through an automated bone scan index (aBSI) (Armstrong et al. 2018). A major issue here is that this would require whole-body ^99m^Tc SPECT imaging, which is usually time-consuming and, therefore, potentially distressing for the patients. We ordinarily use one field of view and do not always have the time or evidence to justify the scans to create numbers for no established foreseeable clinical benefit. However, recent work shows that fast diagnostic whole-body SPECT-CT is possible (Zacho et al. 2017). Three-dimensional viewing might also help spot lesions which would be misclassified in the planar view. But how often does this need to occur to make the cost justifiable? And how plausible is it that we can accurately outline every single lesion in each patient for ongoing quantitative disease assessments?

Since we are still in the early stages of implementation, there are voids of knowledge and experience across different departments, while individuals who do push for change are not always in positions to enact it.

Eagerness surrounding quantification is perhaps based on the simplicity of concept of its appeal: most cases involve focal uptake, whose causes can be non-specific (e.g. pain). The appeal is easy to grasp. But the nature of uptake in bone varies widely and the scope of practice is actually larger than first attributed—many pathologies are packed into the category of ‘bone SPECT’ and quantification might only be useful for a fraction of them, and, as demonstrated by our literature review, evidence significantly varies depending on the application and is not always attached clinical value.

The results of SPECT-CT do not always render an investigation more specific (Van den Wyngaert et al. 2016; Haraldsen et al. 2016). Perhaps ^18^F-NaF is a superior option going forward: PET generally boasts better resolution, lesion contrast, and sensitivity; it only requires one acquisition; and it is already geared up for quantification across the board (Beheshti et al. 2015; Segall et al. 2010; Stauss et al. 2010). However, demand for PET is often high and its investigations are expensive, while data supporting the routine use of bone investigations are relatively scarce.

There is a noticeable drive for SPECT quantification to follow in the footsteps of PET-CT by imitating the implementation of SUV. This drive is understandable, not least for its convenience, but it brushes over the known inaccuracies in PET-CT SUV and acts to eliminate the possibility of implementing alternative metrics which could be more useful. SUV_max_ and SUV_peak_, however, should still be considered strong contenders for quantifying focal uptake (Suh et al. 2016). If SUV_max_ is our most-useful parameter_,_ as Kaneta et al.’s results suggest, understanding of the meaning of extracted numbers across bone scintigraphy investigations is still relatively small (Kaneta et al. 2016). To reduce uncertainties, narrower subsets of condition-specific patients should be recruited, preferably prospectively, to build relevant evidence (e.g. within age, height, weight, or sex-matched cohorts). Cut-off points and thresholds could then be calculated clinically with significantly greater reliability than they are currently.

Generally, more work into the interpretation of numbers is required to understand the quantitative nature of uptake, although this is beginning to emerge (Yamane et al. 2018). But even if the accuracy, precision, and repeatability of data resulting from quantitative bone SPECT can be independently and experimentally validated with respect to standard radioactivity concentrations, the clinical interpretation of a range of results for a chosen method must, at the very least, be understood for the patient’s specific condition. It is not enough to demonstrate statistical significance between areas in a particular investigation: clinical significance is fundamentally more pertinent to the clinical question.

We should continue to pursue evidence on the subject—but not at the cost of expenditure which should be used to support more-evidence-bearing investigations elsewhere. Ideas are more likely to translate into clinical practice if they are cost effective, time-efficient, and beneficial for patients. Many quantitative SPECT applications continue to be introduced but, as shown from our literature search, there is still a scarcity of useful evidence to build a compelling case for the implementation of quantitative bone SPECT in routine clinical practice.

## Conclusion

The aim of quantification should be to optimise the evidence which becomes available to us, enabling us to manage patients more effectively. The potential quantitative bone SPECT holds is undeniable. However, it is still in its infancy. Significant amounts of research and technological improvements are required before it becomes a part of routine clinical practice.

## Abbreviations

ARSAC: Administration of Radioactive Substances Advisory Committee
CT: Computed tomography
DPD: 3,3-dicarboxypropane-1,1-diphosphonate
EANM: European Association of Nuclear Medicine
FDG: Fludeoxyglucose
HMDP: Hydroxymethylene diphosphonate
HDP: Hydroxyethylene diphosphonate
MDP: Methylene diphosphonate
OSEM: Ordered-subset expectation maximisation
PET: Positron emission tomography
PSMA: Prostate-specific membrane antigen
SPECT: Single-photon computed emission tomography
SUV: Standardised uptake value

## References

[CR1] Al-Riyami K, Vöö S, Gnanasegaran G, Pressney I, Meir A, Casey A (2018). The role of bone SPECT/CT in patients with persistent or recurrent lumbar pain following lumbar spine stabilization surgery. Eur J Nucl Med Mol Imaging.

[CR2] Armstrong Andrew J., Anand Aseem, Edenbrandt Lars, Bondesson Eva, Bjartell Anders, Widmark Anders, Sternberg Cora N., Pili Roberto, Tuvesson Helen, Nordle Örjan, Carducci Michael A., Morris Michael J. (2018). Phase 3 Assessment of the Automated Bone Scan Index as a Prognostic Imaging Biomarker of Overall Survival in Men With Metastatic Castration-Resistant Prostate Cancer. JAMA Oncology.

[CR3] Armstrong IS, Hoffmann SA (2016). Activity concentration measurements using a conjugate gradient (Siemens xSPECT) reconstruction algorithm in SPECT/CT. Nucl Med Commun.

[CR4] Bailey DL, Willowson KP (2013). An evidence-based review of quantitative SPECT imaging and potential clinical applications. J Nucl Med.

[CR5] Beck M, Sanders JC, Ritt P, Reinfelder J, Kuwert T (2016). Longitudinal analysis of bone metabolism using SPECT/CT and ^99m^Tc-diphosphono-propanedicarboxylic acid: comparison of visual and quantitative analysis. EJNMMI Res.

[CR6] Beheshti M, Mottaghy FM, Paycha F, Behrendt FFF, Van den Wyngaert T, Fogelman I (2015). ^18^F-NaF PET/CT: EANM procedure guidelines for bone imaging. Eur J Nucl Med Mol Imaging.

[CR7] Buchbender C, Hartung-Knemeyer V, Forsting M, Antoch G, Heusner TA (2013). Positron emission tomography (PET) attenuation correction artefacts in PET/CT and PET/MRI. Br J Radiol.

[CR8] Cachovan M, Vija AH, Hornegger J, Kuwert T (2013). Quantification of 99mTc-DPD concentration in the lumbar spine with SPECT/CT. EJNMMI Res.

[CR9] Chicco A, Lin P, Som S (2015) Assessment and correction of partial volume effect in SPECT/CT. J Intern Med 45.

[CR10] Chirindel A, Alluri KC, Tahari AK, Chaudhry M, Wahl RL, Lodge MA (2015). Liver standardized uptake value corrected for lean body mass at FDG PET/CT: effect of FDG uptake time. Clin Nucl Med.

[CR11] Choi J, Kim JW, Jeon TJ, Lee IJ. The 18F-FDG PET/CT response to radiotherapy for patients with spinal metastasis correlated with the clinical outcomes. Woloschak GE, editor. PLoS One 2018;13:e0204918 10.1371/journal.pone.0204918 PMC6161908 30265736

[CR12] De Laroche R, Simon E, Suignard N, Williams T, Henry M-P, Robin P (2018). Clinical interest of quantitative bone SPECT-CT in the preoperative assessment of knee osteoarthritis. Medicine (Baltimore).

[CR13] Fonager RF, Zacho HD, Langkilde NC, Fledelius J, Ejlersen JA, Haarmark C (2017). Diagnostic test accuracy study of ^18^F-sodium fluoride PET/CT, ^99m^Tc-labelled diphosphonate SPECT/CT, and planar bone scintigraphy for diagnosis of bone metastases in newly diagnosed, high-risk prostate cancer. Am J Nucl Med Mol Imaging.

[CR14] Fraser AORSACNPL (2018). Notes for guidance on the clinical administration of radiopharmaceuticals and use of sealed radioactive sources.

[CR15] Haraldsen A, Bluhme H, Røhl L, Pedersen EM, Jensen AB, Hansen EB (2016). Single photon emission computed tomography (SPECT) and SPECT/low-dose computerized tomography did not increase sensitivity or specificity compared to planar bone scintigraphy for detection of bone metastases in advanced breast cancer. Clin Physiol Funct Imaging.

[CR16] Helyar V, Mohan HK, Barwick T, Livieratos L, Gnanasegaran G, Clarke SEM (2010). The added value of multislice SPECT/CT in patients with equivocal bony metastasis from carcinoma of the prostate. Eur J Nucl Med Mol Imaging.

[CR17] Hetzel M, Arslandemir C, König H-H, Buck AK, Nüssle K, Glatting G (2003). F-18 NaF PET for detection of bone metastases in lung cancer: accuracy, cost-effectiveness, and impact on patient management. J Bone Miner Res.

[CR18] Holman BF, Cuplov V, Millner L, Hutton BF, Maher TM, Groves AM (2015). Improved correction for the tissue fraction effect in lung PET/CT imaging. Phys Med Biol.

[CR19] Huang SC (2000). Anatomy of SUV. Standardized uptake value. Nucl Med Biol.

[CR20] Jarritt PH, Whalley DR, Skrypniuk JV, Houston AS, Fleming JS, Cosgriff PS (2002). UK audit of single photon emission computed tomography reconstruction software using software generated phantoms. Nucl Med Commun.

[CR21] Kaneta T, Ogawa M, Daisaki H, Nawata S, Yoshida K, Inoue T (2016). SUV measurement of normal vertebrae using SPECT/CT with Tc-99m methylene diphosphonate. Am J Nucl Med Mol Imaging..

[CR22] Kim J, Lee H-H, Kang Y, Kim TK, Lee SW, So Y (2017). Maximum standardised uptake value of quantitative bone SPECT/CT in patients with medial compartment osteoarthritis of the knee. Clin Radiol.

[CR23] Kuji I, Yamane T, Seto A, Yasumizu Y, Shirotake S, Oyama M (2017). Skeletal standardized uptake values obtained by quantitative SPECT/CT as an osteoblastic biomarker for the discrimination of active bone metastasis in prostate cancer. European J Hybrid Imaging.

[CR24] López Buitrago DF, Ruiz Botero J, Corral CM, Carmona AR, Sabogal A (2017). Comparison of ^99m^Tc-MDP SPECT qualitative vs quantitative results in patients with suspected condylar hyperplasia. Rev Esp Med Nucl Imagen Mol.

[CR25] Miyaji N, Miwa K, Motegi K, Umeda T, Wagatsuma K, Fukai S (2017). Validation of cross-calibration schemes for quantitative bone SPECT/CT using different sources under various geometric conditions. Nihon Hoshasen Gijutsu Gakkai Zasshi.

[CR26] Nakahara T, Daisaki H, Yamamoto Y, Iimori T, Miyagawa K, Okamoto T (2017). Use of a digital phantom developed by QIBA for harmonizing SUVs obtained from the state-of-the-art SPECT/CT systems: a multicenter study. EJNMMI Res.

[CR27] O'Mahoney E, Murray I (2013). Evaluation of a matched filter resolution recovery reconstruction algorithm for SPECT-CT imaging. Nucl Med Commun.

[CR28] Palmedo H, Marx C, Ebert A, Kreft B, Ko Y, Türler A (2014). Whole-body SPECT/CT for bone scintigraphy: diagnostic value and effect on patient management in oncological patients. Eur J Nucl Med Mol Imaging.

[CR29] Sanderson T, Gear JI, Murray I, Flux G (2015). The impact of background ratios in calibration phantoms on the accuracy of dosimetry for Y-90 DOTATATE. Nucl Med Commun.

[CR30] Schirrmeister H, Glatting G, Hetzel J, Nüssle K, Arslandemir C, Buck AK (2001). Prospective evaluation of the clinical value of planar bone scans, SPECT, and ^18^F-labeled NaF PET in newly diagnosed lung cancer. J Nucl Med.

[CR31] Segall G, Delbeke D, Stabin MG, Even-Sapir E, Fair J, Sajdak R (2010). SNM practice guideline for sodium ^18^F-fluoride PET/CT bone scans 1.0. J Nucl Med.

[CR32] Sher A, Lacoeuille F, Fosse P, Vervueren L, Cahouet-Vannier A, Dabli D (2016). For avid glucose tumors, the SUV peak is the most reliable parameter for [^18^F]FDG-PET/CT quantification, regardless of acquisition time. EJNMMI Res.

[CR33] Stauss J, Hahn K, Mann M, De Palma D (2010). Guidelines for paediatric bone scanning with ^99m^Tc-labelled radiopharmaceuticals and ^18^F-fluoride. Eur J Nucl Med Mol Imaging.

[CR34] Stokke C, Gabiña PM, Solný P, Cicone F, Sandström M, Gleisner KS (2017). Dosimetry-based treatment planning for molecular radiotherapy: a summary of the 2017 report from the Internal Dosimetry Task Force. EJNMMI Phys.

[CR35] Suh MS, Lee WW, Kim Y-K, Yun P-Y, Kim SE (2016). Maximum standardized uptake value of ^99m^Tc hydroxymethylene diphosphonate SPECT/CT for the evaluation of temporomandibular joint disorder. Radiology..

[CR36] Tsujimoto M, Shirakawa S, Teramoto A, Ishiguro I, Nakane K, Ida Y (2018). Fluctuation of quantitative values on acquisition time and the reconstruction conditions in ^99m^Tc-SPECT. Nucl Med Commun.

[CR37] Umeda T, Koizumi M, Fukai S, Miyaji N, Motegi K, Nakazawa S (2018). Evaluation of bone metastatic burden by bone SPECT/CT in metastatic prostate cancer patients: defining threshold value for total bone uptake and assessment in radium-223 treated patients. Ann Nucl Med.

[CR38] Van den Wyngaert T, Strobel K, Kampen WU, Kuwert T, van der Bruggen W, Mohan HK (2016). The EANM practice guidelines for bone scintigraphy. Eur J Nucl Med Mol Imaging.

[CR39] Vija H (2013). White Paper: introduction to xSPECT technology: evolving multi-modal SPECT to become context-based and quantitative.

[CR40] Wang Ruifeng, Duan Xiaoyi, Shen Cong, Han Dong, Ma Junchao, Wu Hulin, Xu Xiaotong, Qin tao, Fan Qiuju, Zhang Zhaoguo, Shi Weihua, Guo Youmin (2018). A retrospective study of SPECT/CT scans using SUV measurement of the normal pelvis with Tc-99m methylene diphosphonate. Journal of X-Ray Science and Technology.

[CR41] Yamane T, Kuji I, Seto A, Matsunari I (2018). Quantification of osteoblastic activity in epiphyseal growth plates by quantitative bone SPECT/CT. Skelet Radiol.

[CR42] Zacho HD, Biurrun Manresa JA, Aleksyniene R, Ejlersen J, Fledelius J, Bertelsen H (2017). Three-minute SPECT/CT is sufficient for the assessment of bone metastasis as add-on to planar bone scintigraphy: prospective head-to-head comparison to 11-min SPECT/CT. EJNMMI Res.

